# Supplementary material for: Identification of New EGFR Inhibitors by Structure-Based Virtual Screening and Biological Evaluation
Source: Int J Mol Sci. 2024 Feb 4;25(3):1887. doi: 10.3390/ijms25031887 (PMC10856253; doi:10.3390/ijms25031887)
Supplement: Supplementary file 1 [file ijms-25-01887-s001.zip › ijms-2785588-SI.pdf]

Identification of New EGFR inhibitors by structure-based virtual screening and biological evaluation

Shuyi Wang[1], Xiaotian, Xu[1], Chuxin Pan, Qian Guo, Qinlan Li, Shanhe Wan, Zhonghuang Li, Jiajie Zhang, Xiaoyun Wu*

Guangdong Provincial Key Laboratory of New Drug Screening, School of Pharmaceutical Sciences, Southern Medical University, Guangzhou 510515, PR China

*Corresponding author:

E-mail: xywu@smu.edu.com (X.-Y. Wu), **Tel.**: +86-20-62789415.

[1]These authors contributed equally to this work.

Table S1 ZINC code and the structure of the selected compounds

Title	ZINC code	structure
**1**	ZINC49691377	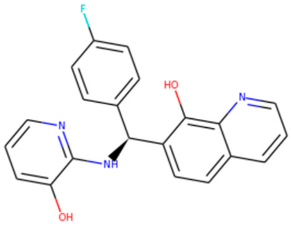
**2**	ZINC09616958	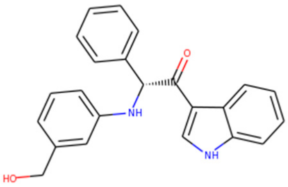
**3**	ZINC09775243	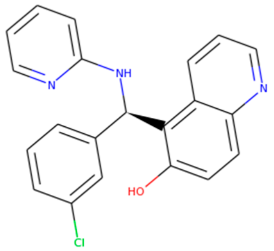
**4**	ZINC10910059	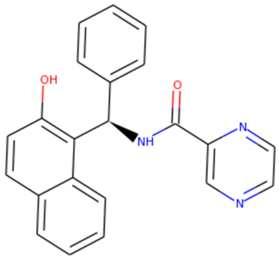

**5**	ZINC89827617	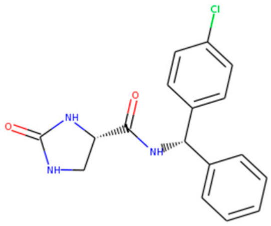
**6**	ZINC22017635	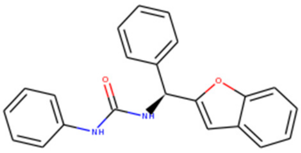
**7**	ZINC53674458	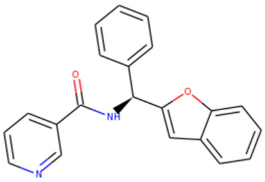
**8**	ZINC69419433	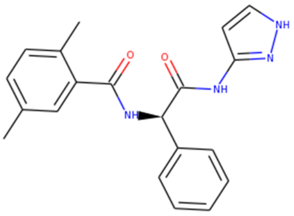
**9**	ZINC15778674	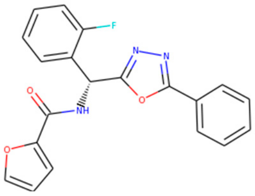
**10**	ZINC29507326	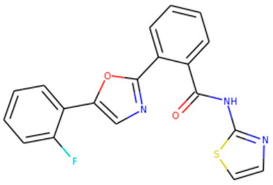
**11**	ZINC05577262	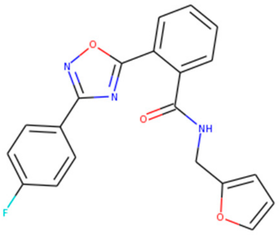
**12**	ZINC03876430	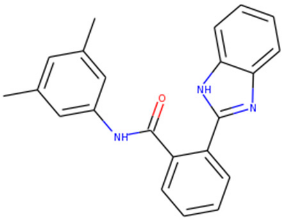

**13**	ZINC18205922	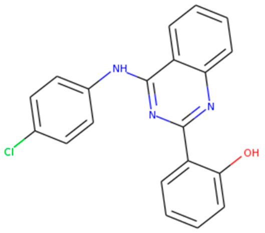
**14**	ZINC00036286	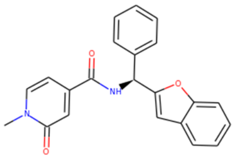
**15**	ZINC20531081	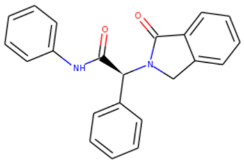
**16**	ZINC89756684	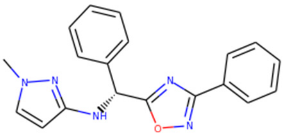
**17**	ZINC01201194	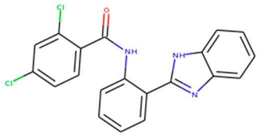
**18**	ZINC01108543	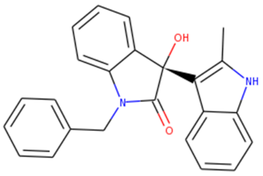
**19**	ZINC13351329	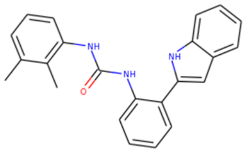
**20**	ZINC04918676	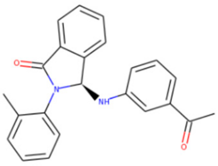
**21**	ZINC04784223	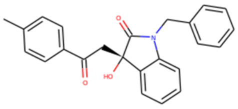

**22**	ZINC43232082	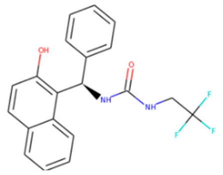
**23**	ZINC00178936	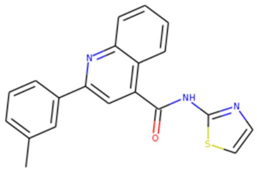

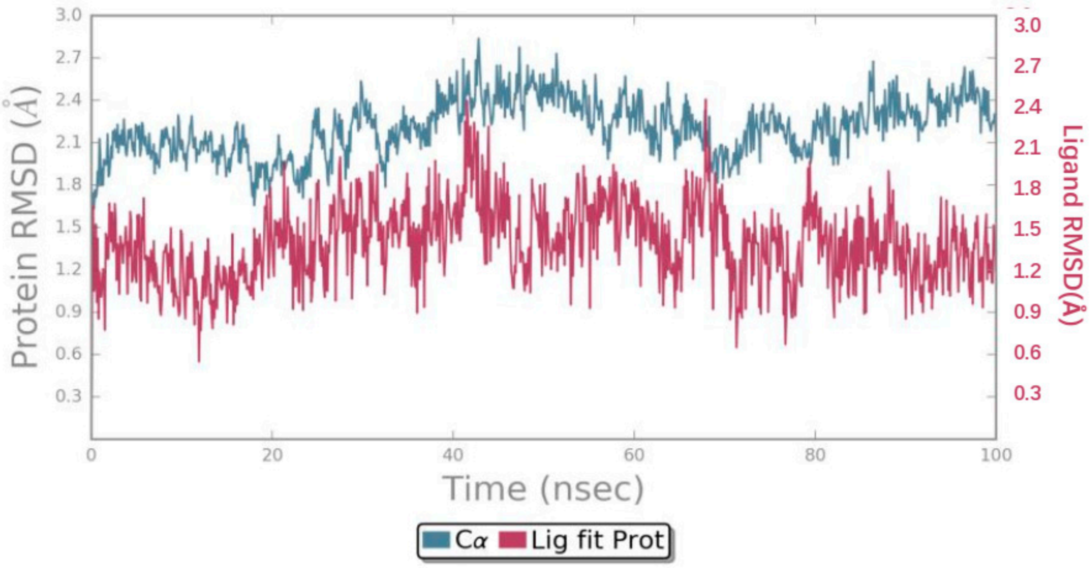

Figure S1. RMSDs of carbon alpha atoms (Å) and ligand atoms (Å) for the co-crystal structure of EAI001 with EGFR during 100 ns MD simulation.

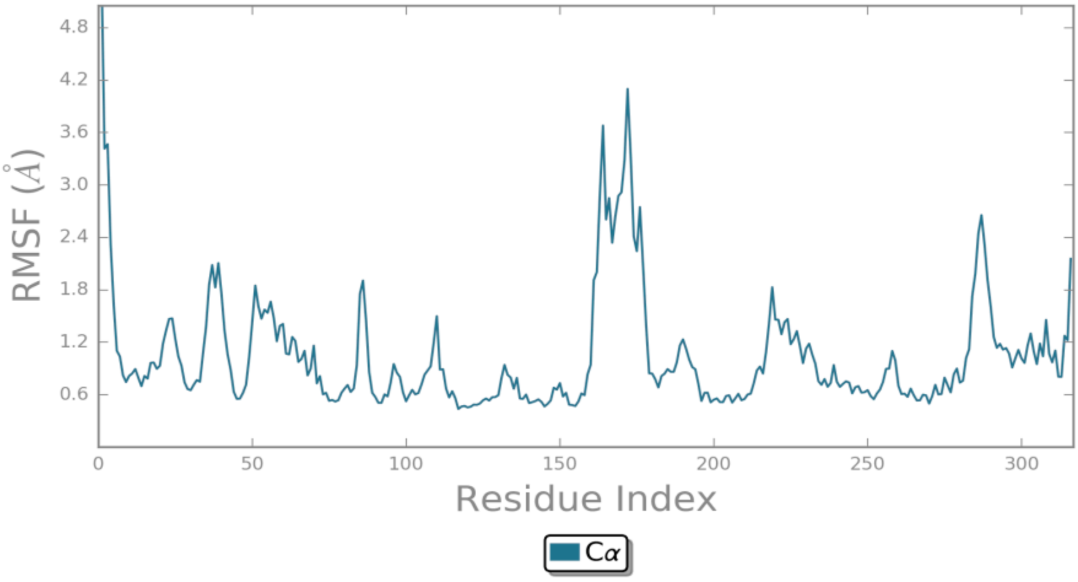

Figure S2. Root mean square fluctuation (RMSF, Å) of carbon alpha for the co-crystal structure of EAI001 with EGFR during 100 ns MD simulation.

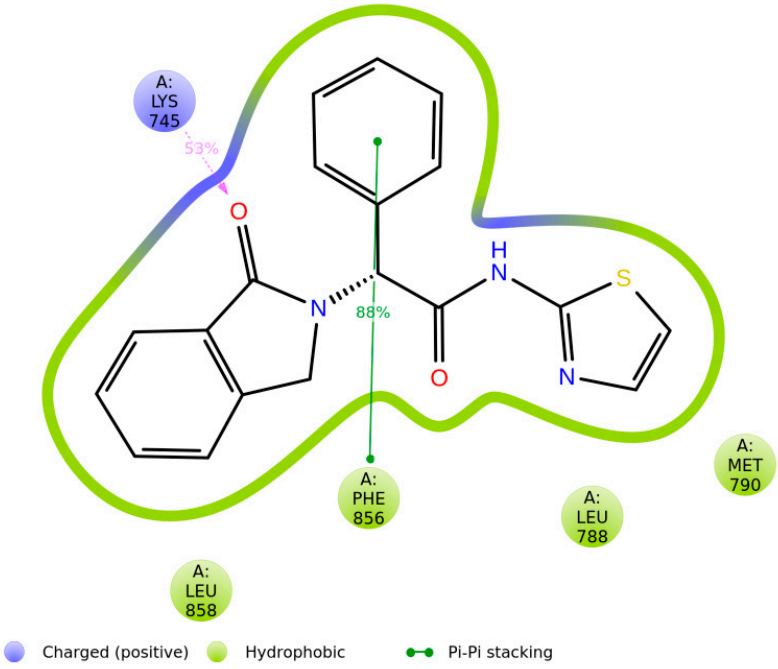

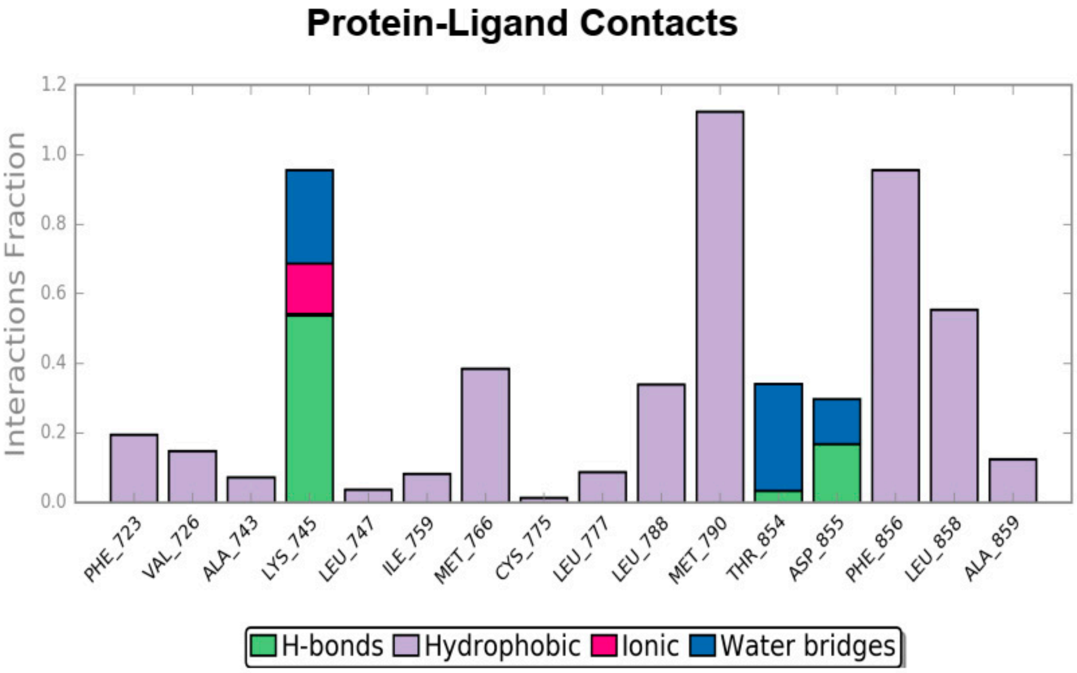

Figure S3. The 2D-interaction diagrams (top) and interaction histograms (bottom) of the co-crystal structure of EAI001 with EGFR during 100 ns MD simulation. Green, purple, red, and blue represent hydrogen bond interactions, hydrophobic interactions, ionic interactions, and water bridges, respectively.

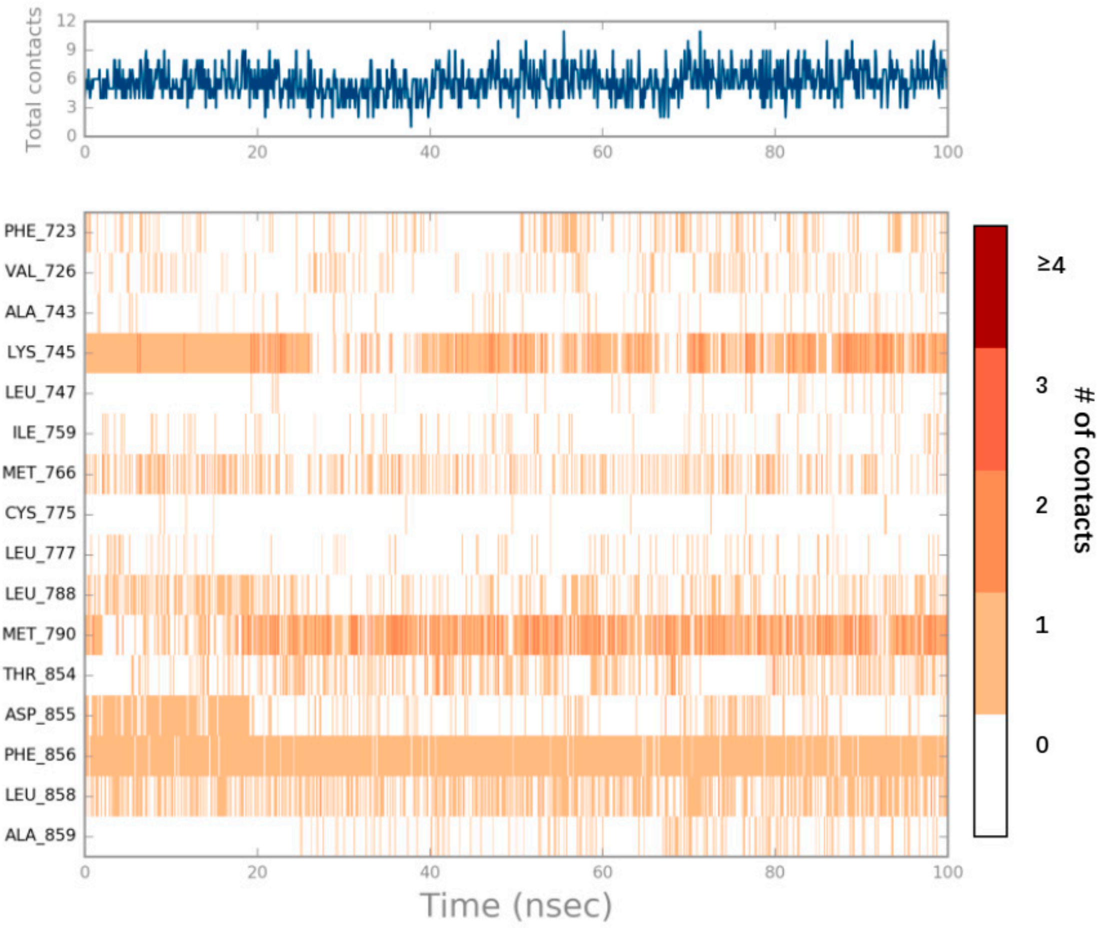

Figure S4. A timeline representation of the interactions and contacts between EAI001 and EGFR during 100 ns MD simulation. The top panel shows the total number of specific contacts the protein makes with the ligand over the course of the trajectory. The bottom panel shows which residues interact with the ligand in each trajectory frame. Some residues make more than one specific contact with the ligand, which is represented by a darker shade of orange, according to the scale to the right of the plot.

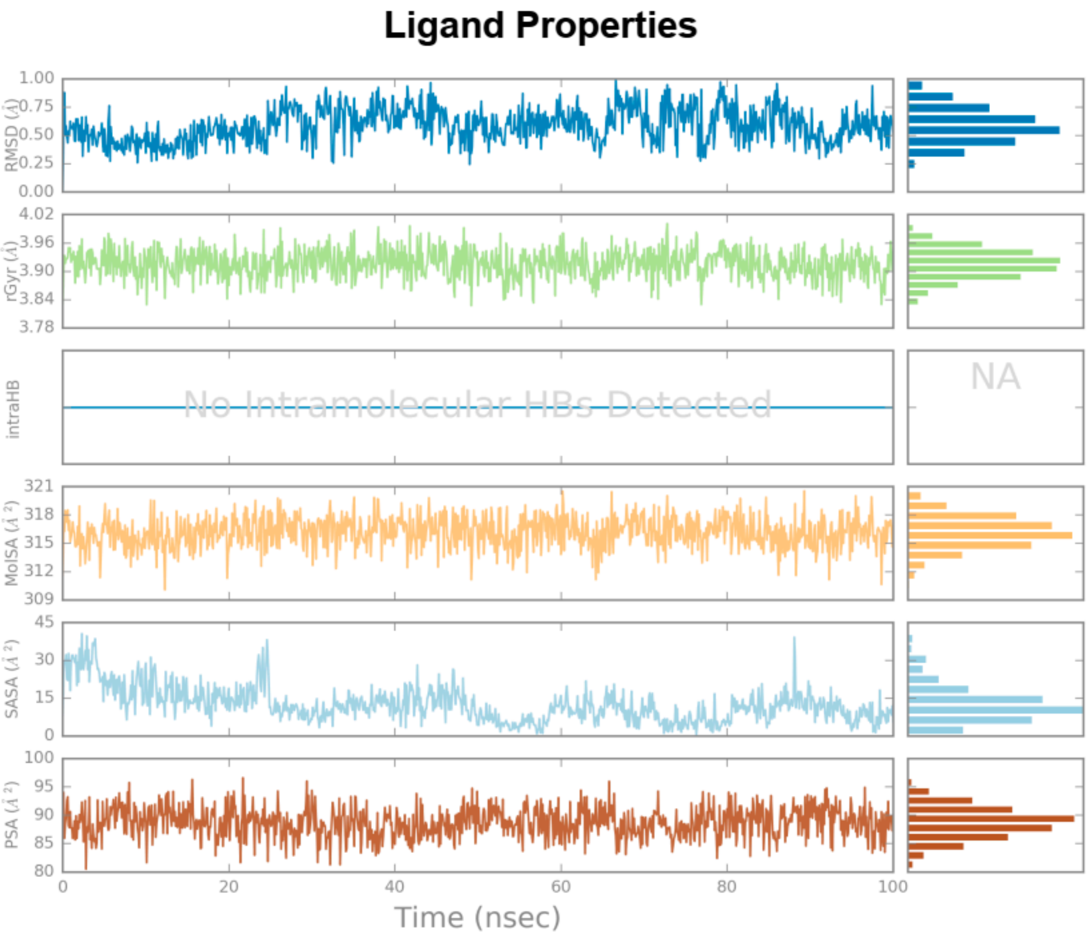

Figure S5. Ligand properties of EAI001 in complex with EGFR throughout 100 ns MD simulation. RMSD: Root mean square deviation of a ligand with respect to the reference conformation. Radius of Gyration (rGyr): Measures the 'extendedness' of a ligand, and is equivalent to its principal moment of inertia. Intramolecular Hydrogen Bonds (intraHB): Number of internal hydrogen bonds (HB) within a ligand molecule. Molecular Surface Area (MolSA): Molecular surface calculation with 1.4 Å probe radius. This value is equivalent to a van der Waals surface area. Solvent Accessible Surface Area (SASA): Surface area of a molecule accessible by a water molecule.Polar Surface Area (PSA): Solvent accessible surface area in a molecule contributed only by oxygen and nitrogen atoms.
